# Association of Maternal Inflammation During Pregnancy With Birth Outcomes and Infant Growth Among Women With or Without HIV in India

**DOI:** 10.1001/jamanetworkopen.2021.40584

**Published:** 2021-12-22

**Authors:** Mehr Shafiq, Jyoti S. Mathad, Shilpa Naik, Mallika Alexander, Su Yadana, Mariana Araújo-Pereira, Vandana Kulkarni, Prasad Deshpande, Nathella Pavan Kumar, Subash Babu, Bruno B. Andrade, Cheng-Shiun Leu, Saltanat Khwaja, Ramesh Bhosale, Aarti Kinikar, Amita Gupta, Rupak Shivakoti

**Affiliations:** 1 Department of Epidemiology, Columbia University Mailman School of Public Health, New York, New York; 2 Department of Medicine, Weill Cornell Medical College, New York, New York; 3 Department of Obstetrics and Gynecology, Byramjee Jeejeebhoy Government Medical College, Pune, India; 4 Byramjee Jeejeebhoy Government Medical College–Johns Hopkins University Clinical Research Site, Pune, India; 5 Instituto Goncalo Moniz, Fundação Oswaldo Cruz, Salvador, Brazil; 6 Multinational Organization Network Sponsoring Translational and Epidemiological Research Initiative, Salvador, Brazil; 7 Faculdade de Medicina, Universidade Federal da Bahia, Salvador, Brazil; 8 National Institutes of Health, National Institute for Research in Tuberculosis, International Center for Excellence in Research, Chennai, India; 9 Curso de Medicina, Faculdade de Tecnologia e Ciências, Salvador, Brazil; 10 Universidade Salvador, Laureate Universities, Salvador, Brazil; 11 Curso de Medicina, Escola Bahiana de Medicina e Saúde Pública, Salvador, Brazil; 12 Department of Biostatistics, Columbia University Mailman School of Public Health, New York; 13 Department of Paediatrics, Byramjee Jeejeebhoy Government Medical College, Pune, India; 14 Department of Medicine, Johns Hopkins University School of Medicine, Baltimore, Maryland

## Abstract

**Importance:**

The association of elevated levels of specific inflammatory markers during pregnancy with adverse birth outcomes and infant growth could indicate pathways for potential interventions.

**Objective:**

To evaluate whether higher levels of certain inflammatory markers during pregnancy are associated with preterm birth (PTB), low birth weight (LBW), and infant growth deficits.

**Design, Setting, and Participants:**

In this cohort study of pregnant women with or without HIV, 218 mother-infant pairs were followed up from pregnancy through 12 months post partum from June 27, 2016, to December 9, 2019. Pregnant women aged 18 to 40 years and between 13 and 34 weeks of gestation who were receiving antenatal care were enrolled in a cohort stratified by HIV status; sampling was based on convenience sampling from women receiving antenatal care at Byramjee Jeejeebhoy Government Medical College.

**Exposures:**

Levels of multiple circulating inflammation markers during the third trimester of pregnancy.

**Main Outcomes and Measures:**

The primary study outcome was PTB (<37 weeks’ gestation). Secondary outcomes were LBW (<2500 g) and repeated measures (delivery; 6 weeks post partum; and 3, 6, and 12 months post partum using multivariable generalized linear models) of infant growth outcomes (length-for-age, weight-for-age, and weight-for-length *z* scores).

**Results:**

The median age of the 218 women at enrollment was 23 years (IQR, 21-27 years). In multivariable models, higher pregnancy levels of interleukin 17A were associated with increased odds of both PTB (adjusted odds ratio [aOR], 2.62; 95% CI, 1.11-6.17) and LBW (aOR, 1.81; 95% CI, 1.04-3.15). Higher levels of interleukin 1β were associated with increased PTB (aOR, 1.47; 95% CI, 1.15-1.89) and infant growth deficits (lower length-for-age *z* score: adjusted β = −0.10; 95% CI, −0.18 to −0.01; lower weight-for-age *z* score: adjusted β = −0.07; 95% CI, −0.14 to 0.001).

**Conclusions and Relevance:**

This study suggests that increased levels of certain systemic inflammatory markers, including interleukin 1β and interleukin 17A, during pregnancy were associated with adverse birth outcomes and infant growth deficits. Future studies should evaluate whether potential interventions to modulate specific inflammatory pathways during pregnancy could improve birth outcomes and infant growth.

## Key Points

**Question:**

Is elevated inflammation in pregnant women with or without HIV associated with adverse birth outcomes and infant growth deficits?

**Findings:**

In this cohort study of pregnant women in Pune, India, higher levels during pregnancy of interleukin 17A were associated with increased odds of both preterm birth and low birth weight. Higher levels of interleukin 1β were associated with increased preterm birth and infant growth deficits.

**Meaning:**

This study suggests that elevated inflammation during pregnancy is associated with adverse birth outcomes and infant growth deficits, and future studies should test whether modulating specific inflammatory pathways could reduce adverse birth outcomes and growth deficits.

## Teaser

This cohort study evaluates whether higher levels of certain inflammatory markers during pregnancy are associated with preterm birth, low birth weight, and infant growth deficits among women with or without HIV in India.

## Introduction

Pregnancy is characterized by major changes in maternal immunity. Trimester-specific changes have also been observed, with an immunosuppressive and anti-inflammatory profile in midpregnancy to late pregnancy compared with a more proinflammatory profile during labor.^1,2,3^ Alterations to this profile have been associated with adverse pregnancy and birth outcomes; for example, increased systemic levels of proinflammatory biomarkers (such as interleukin 6 [IL-6], tumor necrosis factor, IL-1β, and C-reactive protein) during midpregnancy to late pregnancy were associated with preterm birth (PTB).^4^ However, there are limited data on whether other systemic biomarkers of inflammation, such as those associated with gut integrity, monocyte activation, or helper T (T_H_) cell responses (eg, T_H_2 or T_H_17), are also associated with PTB.

Data are also lacking on how maternal levels of these inflammatory markers are associated with infant birth weight despite low birth weight (LBW) being another common and clinically significant adverse birth outcome, especially in resource-limited settings. Furthermore, despite data supporting the association of perinatal factors with health outcomes during childhood and adulthood within the developmental origins of health and disease framework,^5^ studies have not examined whether and how maternal inflammation during pregnancy is associated with infant growth outcomes. For instance, it is not known whether higher maternal levels of specific systemic inflammatory markers during pregnancy are associated with infant length and weight measures related to stunting, wasting, and being underweight. Understanding the association of maternal inflammatory responses during pregnancy with birth and infant outcomes could potentially help identify interventions (eg, anti-inflammatory agents) that could reduce adverse birth outcomes and infant growth deficits.^6^

To address these gaps in research, we measured inflammatory markers, informing on aspects of general inflammation and acute phase response, inflammasome activation, gut integrity, monocyte activation, and T_H_ responses in pregnant women from a cohort study in India. We then assessed the association of these markers with adverse birth outcomes (PTB and LBW) and infant growth (length-for-age *z* score [LAZ], weight-for-age *z* score [WAZ], and weight-for-length *z* score [WLZ]).

## Methods

### Study Design and Population

We conducted a longitudinal cohort study of pregnant women (PRACHITi [Pregnancy Associated Changes in Tuberculosis Immunology] study) in Pune, India, from June 27, 2016, to December 9, 2019.^7^ We enrolled adult pregnant women aged 18 to 40 years and between 13 and 34 weeks of gestation (confirmed by early pregnancy ultrasonography) who were receiving antenatal care at Byramjee Jeejeebhoy Government Medical College, a tertiary care hospital that serves primarily low-income populations and is a referral center for HIV care. The study excluded pregnant women with active tuberculosis or severe anemia at entry, as well as women who were taking antibiotics for more than 14 days, had a history of an autoimmune or immunosuppressive disease, or were taking immunosuppressive medication. As the primary aim of the PRACHITi study was to compare immune responses during pregnancy by HIV status, women were enrolled in a cohort (N = 218) stratified by HIV status (69 HIV positive and 149 HIV negative).^8^ Sample size was determined based on the PRACHITi study’s goals. Sampling within each stratum was based on convenience sampling of those who met the eligibility criteria.

This study received approval from the institutional review boards of The Johns Hopkins University, Columbia University, Weill Cornell Medicine, and Byramjee Jeejeebhoy Government Medical College. Written informed consent was obtained from all mothers. All guidelines for human experimentation from the US Department of Health and Human Services were followed. This study followed the Strengthening the Reporting of Observational Studies in Epidemiology (STROBE) reporting guideline for observational studies.

### Data Collection and Laboratory Procedures

We collected sociodemographic information and clinical data from study participants at enrollment. The gestational age of the mothers was determined by ultrasonography performed in early pregnancy. Follow-up visits were conducted during the third trimester (for those enrolled in the second trimester), at delivery, 6 weeks post partum, and 3, 6, and 12 months post partum. Gestational age at delivery, along with infant length and weight at each visit, were recorded thereafter. Further details on data collection can be found in the eMethods in the Supplement. During these visits, heparin plasma samples were extracted from maternal blood samples and stored until further assessment of immune biomarkers. As detailed previously,^7^ single-plex immunoassays were performed on third-trimester plasma samples according to the manufacturer’s (R&D Systems) directions for soluble CD163, soluble CD14 (sCD14), intestinal fatty acid–binding protein, C-reactive protein, alpha 1-acid glycoprotein, and interferon β. Multiplex immunoassays (Luminex assays; R&D Systems) measuring interferon γ, IL-1β, IL-6, IL-13, IL-17A, and tumor necrosis factor were also performed on these samples. These immune biomarkers were chosen based on their importance to birth outcomes and HIV.^9^

### Statistical Analysis

We generated descriptive statistics of sample characteristics for the overall sample as well as by the PTB and LBW status of infants. Preterm birth was defined as birth prior to 37 weeks of gestation, and LBW was defined as birth weight less than 2500 g. The Fisher exact test was used to assess differences in study population characteristics by PTB and LBW for categorical variables, and the Wilcoxon rank sum test was used for continuous variables owing to the violation of normality assumption for the *t* test. All *P* values were from 2-sided tests, and results were deemed statistically significant at *P* < .05.

We compared median levels of each inflammatory marker during the third trimester between women with PTB and women with term births using the Wilcoxon rank sum test. All inflammatory markers were transformed to the log_2_ scale to approximate normality. To evaluate the association between each inflammatory marker at the third trimester and birth outcomes (ie, PTB [primary outcome] and LBW [secondary and exploratory outcome]), we used logistic regression models with PTB or LBW as separate binary outcome variables. Multivariable model 1 adjusted for maternal age, mid–upper arm circumference (MUAC, a more reliable indicator of maternal nutritional status during pregnancy),^10^ HIV status, parity, smoking, and history of PTB. Multivariable model 2 additionally adjusted for maternal educational level, anemia, and latent tuberculosis infection (LTBI) status.

To evaluate the association between each inflammatory marker at the third trimester and infant growth, we first computed 3 different variables at each time point for LAZ, WAZ, and WLZ using the World Health Organization child growth standards.^11^ We used a generalized linear model with an identity-link function to assess the association of inflammation with infant LAZ, WAZ, and WLZ (separate analysis for each continuous infant growth outcome variable). Multivariable model 1 adjusted for maternal age, MUAC, HIV status, parity, and smoking. Multivariable model 2 additionally adjusted for maternal educational level, anemia, and LTBI. We used a generalized estimating equation method with an exchangeable working correlation matrix and a robust variance estimator to account for the within-individual correlation owing to repeated outcome measures of infant growth at multiple time points of 0 (time of delivery) and 3, 6, and 12 months.

We also conducted exploratory analyses, using similar approaches, to assess whether the association of inflammatory markers with birth outcomes and infant growth differed by strata of HIV infection status. Our analyses for this study are focused on hypothesis generation, and therefore we report and interpret effect estimates and 95% CIs. All analyses were conducted in SAS software, version 9.4 (SAS Institute Inc).

## Results

The median age of the 218 women at enrollment during pregnancy was 23 years (IQR, 21-27 years) (Table). A total of 73 of 216 women (34%) reported a monthly income below India’s poverty line of ₹10 255 Indian rupees (US $138.19),^12^ and 52 (24%) reported having an educational level of primary school or less. A total of 62 women (28%) were undernourished, defined as MUAC less than 23 cm^10^; 193 women (89%) were nonsmokers; and 18 women (8%) had a history of PTB. Based on the stratified design of the parent study, 69 women (32%) had HIV infection. All women with HIV were receiving antiretroviral therapy, and 52 of them (75%) were receiving efavirenz-based regimens. Twenty-five of the women (12%) in this cohort gave birth to PTB infants. The study population characteristics did not differ significantly by PTB status.

**Table.** Characteristics of the Study Population During Their Third Trimester by Preterm Birth Status

Characteristic	No. (%)^a^			*P* value^b^
	Overall (N = 218)	Preterm births (n = 25 [12%])	Term births (n = 193 [89%])	
Age, median (IQR), y	23 (21-27)	23 (22-26)	23 (20-27)	.43
Monthly income, No./total No. (%)				
≤ ₹10 255 (US $138.19)	73/216 (34)	12/24 (50)	61/192 (32)	.11
> ₹10 255 (US $138.19)	143/216 (66)	12/24 (50)	131/192 (68)	
Educational level				
None to primary	52 (24)	8 (32)	44 (23)	.57
Middle school to high school	139 (64)	15 (60)	124 (64)	
After high school	27 (12)	2 (8)	25 (13)	
Mid–upper arm circumference, cm				
<23.0	62 (28)	10 (40)	52 (27)	.23
23.0-30.5	142 (65)	15 (60)	127 (66)	
>30.5	14 (6)	0	14 (7)	
Smoking status				
Yes	25 (12)	4 (16)	21 (11)	.50
No	193 (89)	21 (84)	172 (89)	
History of preterm birth				
Yes	18 (8)	4 (16)	14 (7)	.13
No	200 (92)	21 (84)	179 (93)	
HIV				
Yes	69 (32)	8 (32)	61 (32)	.99
No	149 (68)	17 (68)	132 (68)	

^a^ Percentages may not total 100% because of rounding.

^b^ *P* values were calculated using the Fisher exact test for categorical variables and the Wilcoxon rank sum test for continuous variables to determine the difference between mothers who had preterm deliveries and mothers who had term deliveries.

### Maternal Inflammation During Pregnancy and Birth Outcomes

#### Preterm Birth

The median gestational age at which we measured the biomarkers was 29.3 weeks (IQR, 28.5-30.4 weeks). We compared the median log_2_-transformed levels of third-trimester inflammatory markers by birth status (ie, PTB vs term births) using the Wilcoxon rank sum test (eFigure in the Supplement). Median log_2 _IL-1β and IL-17A levels were significantly higher in women with PTB compared with women with term births (IL-1β, 4.55 vs 2.40 pg/mL; *P* = .002; IL-17A, 2.56 vs 2.27 pg/mL; *P* = .008).

**Figure 1.**
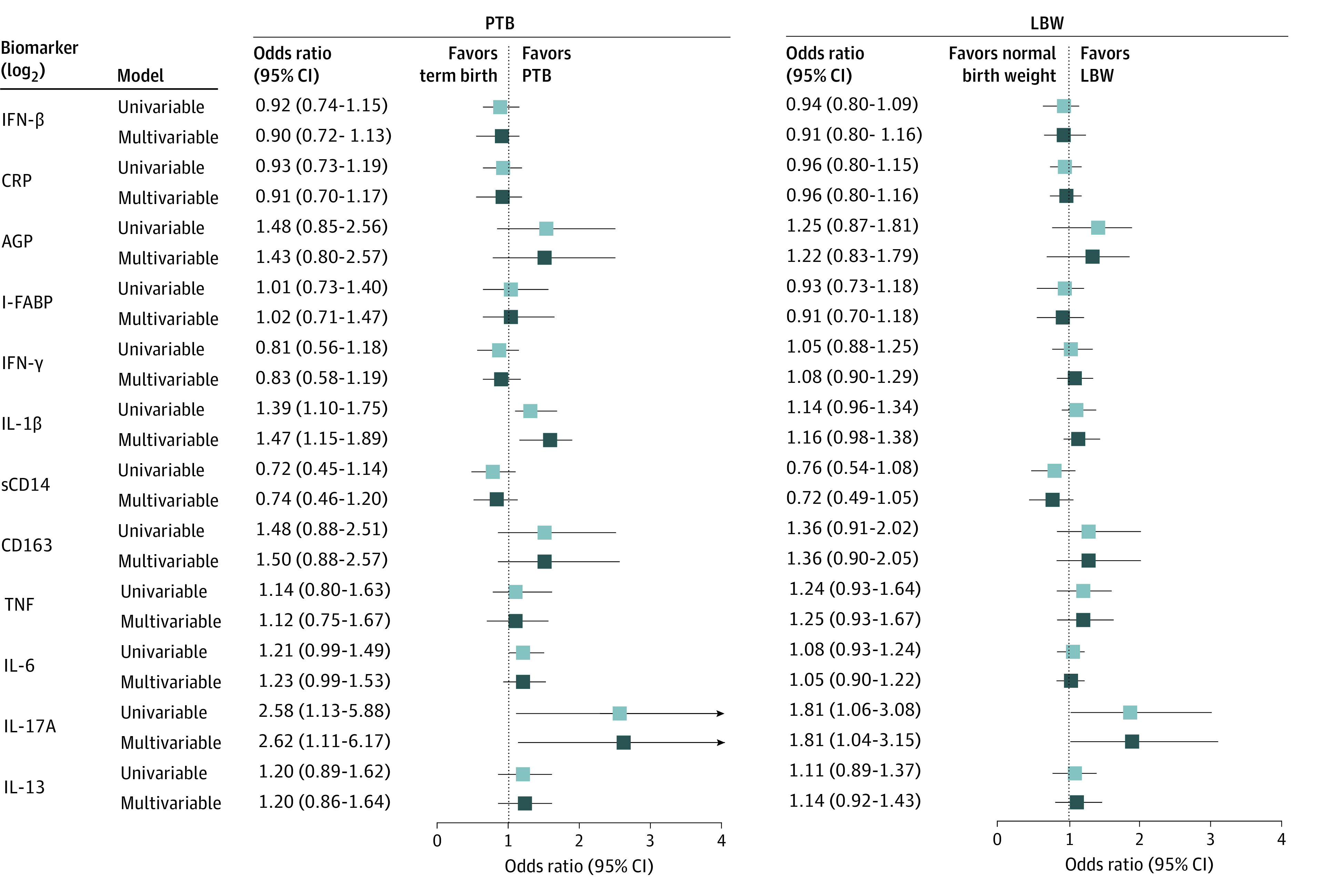
Association of Inflammatory Markers With Preterm Birth (PTB) and Low Birth Weight (LBW) The odds of PTB and LBW per increase in log_2_ concentrations (and 95% CIs) of each inflammation marker (third trimester). Only the univariable model and multivariable model 1 are shown here. Multivariable model 1 adjusted for maternal age, mid–upper arm circumference, HIV status, parity, smoking, and history of PTB. AGP indicates alpha 1-acid glycoprotein; CRP, C-reactive protein; I-FABP, intestinal fatty acid–binding protein; IFN, interferon; IL, interleukin; sCD14, soluble CD14; and TNF, tumor necrosis factor.

We used univariable (N = 218) and multivariable (n = 211) logistic regression models to assess the association between inflammation during pregnancy and PTB. In univariable models, higher levels of IL-1β (odds ratio [OR], 1.39; 95% CI, 1.10-1.75), IL-6 (OR, 1.21; 95% CI, 0.99-1.49), and IL-17A (OR, 2.58; 95% CI, 1.13-5.88) were associated with increased odds of PTB (Figure 1). Similar results were observed for IL-1β and IL-17A in multivariable models adjusting for age, MUAC, smoking, HIV status, parity, and history of PTB (IL-1β: adjusted OR [aOR], 1.47; 95% CI, 1.15-1.89; IL-17A: aOR, 2.62; 95% CI, 1.11-6.17). Similar results were observed in models further adjusting for anemia, educational level, and LTBI for IL-1β (aOR, 1.52; 95% CI, 1.15-2.01) and IL-17A (aOR, 2.36; 95% CI, 0.99-5.64) (eTable 1 in the Supplement). Furthermore, we also had data on second-trimester inflammatory markers from a smaller subset of these women (n = 166) and saw a similar association of IL-1β (aOR, 1.41; 95% CI, 1.02-1.96) and IL-17A (aOR, 4.65; 95% CI, 1.39-15.55), measured using the mean of second-trimester and third-trimester values, with PTB.

#### Low Birth Weight

Data on birth weight and inflammatory markers were available from 213 pregnant women. Thirty percent (n = 64) of the infants in this cohort were born with LBW; the study population characteristics did not differ significantly by LBW status apart from a higher proportion of undernourished women among those who had LBW infants than among those who had normal-weight infants (25 of 64 [39%] vs 34 of 149 [23%]; *P* = .05) (eTable 2 in the Supplement).

We used univariable and multivariable logistic regression models to assess the association between inflammation during pregnancy and LBW. Maternal IL-17A levels were positively associated with LBW in univariable models (OR, 1.81; 95% CI, 1.06-3.08) (Figure 1). When adjusting for age, MUAC, smoking, HIV status, parity, and history of PTB, similar results were observed for the association of IL-17A levels with LBW (aOR, 1.81; 95% CI, 1.04-3.15). Results for IL-17A levels were also statistically significant when further adjusting for anemia, education, and LTBI (aOR, 1.96; 95% CI, 1.09-3.49) (eTable 1 in the Supplement) and in models using the mean of second-trimester and third-trimester values (n = 162) (aOR, 2.30; 95% CI, 1.18-4.49).

### Maternal Inflammation During Pregnancy and Infant Growth

#### Length-for-Age *z* Score

**Figure 2.**
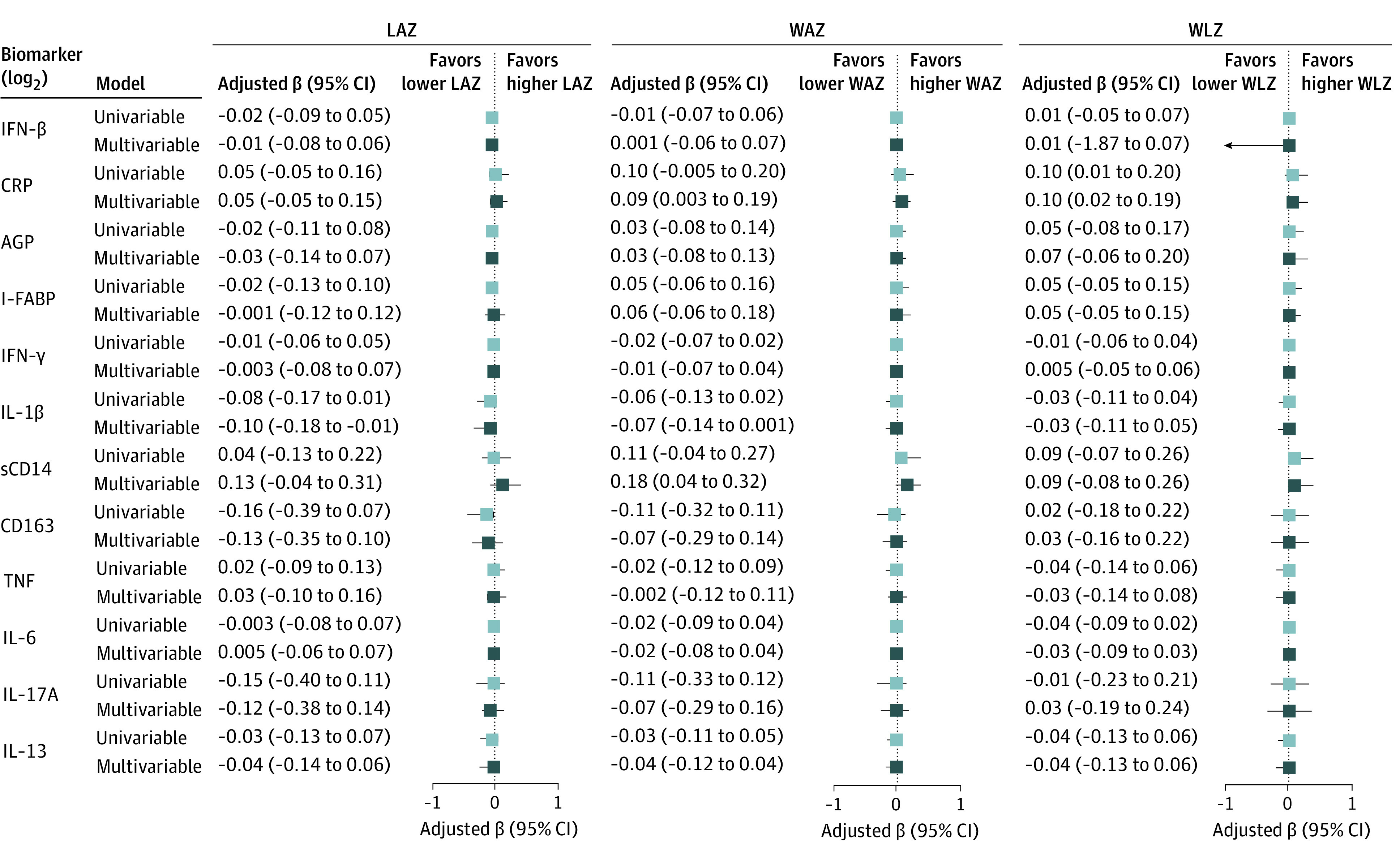
Association of Inflammatory Markers With Infant Growth The mean increase in length-for-age *z* score (LAZ), weight-for-age *z* score (WAZ), and weight-for-length *z* score (WLZ) over the time points of delivery, 6 weeks, and 3 months, 6 months, and 12 months post partum are shown per increase in log_2_ concentrations (and 95% CIs) of each inflammation marker (third trimester). The univariable model and multivariable model 1 are shown here. Multivariable model 1 adjusted for maternal age, mid–upper arm circumference, HIV status, parity, and smoking. AGP indicates alpha 1-acid glycoprotein; CRP, C-reactive protein; I-FABP, intestinal fatty acid–binding protein; IFN, interferon; IL, interleukin; sCD14, soluble CD14; and TNF, tumor necrosis factor.

Follow-up data for the first year of the infant’s life that were needed to calculate LAZ, WAZ, and WLZ were available for 205 infants. We used univariable and multivariable generalized linear models with the generalized estimating equation method to assess the association between inflammation during the third trimester and LAZ during the first year of the infant’s life. Increased levels of maternal IL-1β were associated with a mean decrease in LAZ in univariable (β = −0.08; 95% CI, −0.17 to 0.01) and multivariable (adjusted β = −0.10; 95% CI, −0.18 to −0.01) models when adjusting for maternal age, MUAC, HIV status, parity, and smoking (model 1) (Figure 2). Similar results were observed in models that further adjusted for anemia, educational level, and LTBI (adjusted β = −0.11; 95% CI, −0.21 to −0.02) (eTable 3 in the Supplement). On the other hand, increased levels of sCD14 were associated with a mean increase in LAZ in model 1 (adjusted β = 0.13; 95% CI, −0.04 to 0.31) (Figure 2).

#### Weight-for-Age *z* Score

We used the same generalized linear models with the generalized estimating equation approach to assess the association between inflammatory markers and WAZ during the first year of the infant’s life. Similar to the LAZ results, IL-1β was negatively associated with WAZ (adjusted β = −0.07; 95% CI, −0.14 to 0.001 in multivariable model 1), whereas sCD14 was positively associated with WAZ (adjusted β = 0.18; 95% CI, 0.04-0.32 in multivariable model 1) (Figure 2). Furthermore, C-reactive protein was also positively associated with WAZ (adjusted β = 0.09; 95% CI, 0.003-0.19 in multivariable model 1). Results were similar for IL-1β (adjusted β = −0.08; 95% CI, −0.16 to −0.003), sCD14 (adjusted β = 0.20; 95% CI, 0.05-0.35), and C-reactive protein (adjusted β = 0.09; 95% CI, 0.004-0.18) in multivariable model 2 (eTable 3 in the Supplement).

#### Weight-for-Length *z* Score

For WLZ, only maternal C-reactive protein was positively associated with WLZ during the first year of life. This association was observed in the univariable model (β = 0.10; 95% CI, 0.01-0.20), multivariable model 1 (adjusted β = 0.10; 95% CI, 0.02-0.19) (Figure 2), and multivariable model 2 (adjusted β = 0.10; 95% CI, 0.02-0.19) (eTable 3 in the Supplement).

### Exploratory Analyses of Birth Outcomes and Infant Growth by HIV Status

Although our power was limited to assess whether HIV status was associated with inflammation and birth outcomes, exploratory analyses suggested that IL-1β was associated with PTB in both women with HIV (aOR, 1.88; 95% CI, 1.07-3.31) and women without HIV (aOR, 1.32; 95% CI, 0.98-1.79).

Our results on the association between higher maternal sCD14 levels and higher LAZ and WAZ were surprising. An analysis from this cohort had noted that sCD14 levels were significantly higher among pregnant women with HIV compared with those without HIV.^8^ Thus, based on prior literature that suggests that infants of mothers with HIV have growth deficits at birth, we hypothesized that these results could be partly explained by maternal HIV status. In stratified analysis, in which the positive association of sCD14 with LAZ (adjusted β = 0.42; 95% CI, 0.11-0.73) and WAZ (adjusted β = 0.32; 95% CI, 0.06-0.58) was confirmed, this hypothesis was supported and observed only among mothers with HIV.

## Discussion

In our maternal-infant cohort study from Pune, India, higher levels of various inflammatory markers during pregnancy were independently associated with adverse birth outcomes and infant growth deficits. For example, higher levels of IL-1β during pregnancy were associated with increased PTB and growth deficits, and IL-17A was positively associated with PTB and LBW. These results suggest a need for future studies to test whether modulating specific inflammatory pathways (eg, those associated with the inflammasome pathway or the T_H_17 pathway) could be associated with birth outcomes and growth deficits. If these findings are confirmed, future studies should identify and test such an intervention for improved maternal-infant health outcomes.

In our study, we observed a positive association of high maternal IL-1β levels with PTB, which is in line with existing literature.^3,13^ Interleukin 1β is a cytokine involved in inducing systemic and local immune responses to pathogens, and it does so by enhancing transcription or messenger RNA stability of other proinflammatory genes. This infection-induced proinflammatory environment is consequently shown to be associated with preterm labor.^13^ In addition, IL-1β can also increase prostaglandin levels, which could be associated with increased myometrial contractions and eventual preterm labor. These results were also confirmed by our exploratory analyses that showed that higher levels of IL-1β were positively associated with PTB in women with HIV and those without HIV. In addition, maternal IL-1β levels were associated with the infant growth indicators LAZ and WAZ, in which higher levels of this cytokine were associated with less infant growth. Given that there is limited literature on the association with inflammation during pregnancy and infant growth, to our knowledge, this finding with IL-1β is novel and warrants further confirmation. Although inflammatory markers are known to reduce levels of insulinlike growth factor 1,^14^ which is associated with linear growth, further research into the potential mechanisms is needed.

Levels of IL-17A were associated with both PTB and LBW. Interleukin 17A, a cytokine produced by T_H_17 and other immune cells, plays a crucial role in the defense against various microbial pathogens. While the reasons for increased PTB with IL-17A are unclear, higher levels of IL-17A associated with excess inflammation can result in tissue damage. In fact, limited data suggest that IL-17A in the feto-maternal interface could be associated with PTB,^15^ and future studies should evaluate whether and how circulating levels of IL-17A as measured in this study are associated with PTB. Similar associations were also observed for IL-17A and LBW, another novel association observed by our study. Whether this association is being driven by concurrent PTB needs further assessment. Overall, IL-17A levels could have potential prognostic utility for PTB and LBW, and if this association is causal, interventions to target IL-17A could hypothetically reduce adverse birth outcomes.

Our results also showed a positive association between maternal sCD14 levels and higher LAZ or WAZ. As this result was surprising, we hypothesized that this result could be partly explained by maternal HIV status. More specifically, prior analysis from this cohort had noted that pregnant mothers with HIV had higher levels of sCD14.^8^ Thus, we conducted a stratified analysis for which our results showed that this association was true only in women with HIV. This finding suggests that infants from mothers with higher sCD14 levels start with low LAZ and WAZ at birth and then “catch up” over time. If future studies confirm these results, it would indicate that, while infants born to HIV-infected mothers with high sCD14 levels are likely to be born with growth deficits, they are able to partly recover these deficits through the first year of life.

This study addresses several gaps in the literature related to maternal inflammation and infant health outcomes. Our results, particularly the associations of maternal inflammation during pregnancy with infant growth outcomes, are novel. Furthermore, this study examines multiple inflammatory markers, providing insights into different inflammatory pathways associated with general inflammation, monocyte activation, gut integrity, and inflammasome activation. As most existing research on some of these inflammatory markers and PTB have been conducted in Western countries, our study provides valuable data from India with its unique immune, metabolic,^16^ and nutritional profile.^17^

### Limitations

There are some limitations to this study. The sample size was limited with respect to PTB outcomes and particularly the stratified analyses. Larger studies with formal interaction analyses may be required to highlight how these findings differ by relevant effect modifiers. Our cohort had a high percentage of women with HIV owing to the parent study design, and 28% were undernourished; therefore, our results may not be generalizable to other populations with different characteristics. Additionally, our results associated with HIV largely represent individuals receiving efavirenz-based antiretroviral therapy regimens, and we do not know the generalizability to populations receiving other antiretroviral therapies. Given that we included mothers up to 34 weeks’ gestation, the possibility of selection bias against mothers who delivered before they could be enrolled in the study cannot be discounted. Our findings associated with the secondary outcomes of LBW and growth outcomes were exploratory and will need to be confirmed in future studies. In addition, there may be some unmeasured or unknown confounders that could potentially explain the observed associations. Despite these limitations, our results suggest that specific inflammatory markers during pregnancy are associated with adverse birth outcomes and early infant growth.

## Conclusions

This cohort study found that increased levels of certain inflammatory markers, particularly IL-1β and IL-17A, during pregnancy were associated with adverse birth outcomes and infant growth deficits. If future studies confirm these associations, the mechanisms by which these biomarkers are associated with these outcomes need further study to evaluate whether potential interventions could improve birth outcomes and infant growth.

## Notes

Supplement.
eMethods.
**eFigure.** Levels of Third Trimester Inflammatory Markers by Preterm Birth Status (N = 218) **eTable 1.** Association of Inflammatory Markers With PTB and LBW (Multivariable Model II) **eTable 2.** Characteristics of the Study Population During Their Third Trimester by Low Birth Weight Status (N = 213) **eTable 3.** Association of Inflammatory Markers With LAZ, WAZ, and WLZ (Multivariable Model II)
