# Supplementary material for: Association of Maternal Inflammation During Pregnancy With Birth Outcomes and Infant Growth Among Women With or Without HIV in India
Source: JAMA Netw Open. 2021 Dec 22;4(12):e2140584. doi: 10.1001/jamanetworkopen.2021.40584 (PMC8696571; doi:10.1001/jamanetworkopen.2021.40584)
Supplement: Supplement. — eMethods. eFigure. Levels of Third Trimester Inflammatory Markers by Preterm Birth Status (N = 218) eTable 1. Association of Inflammatory Markers With PTB and LBW (Multivariable Model II) eTable 2. Characteristics of the Study Population During Their Third Trimester by Low Birth Weight Status (N = 213) eTable 3. Association of Inflammatory Markers With LAZ, WAZ, and WLZ (Multivariable Model II) [file jamanetwopen-e2140584-s001.pdf]

Supplementary Online Content

Shafiq M, Mathad JS, Naik S, et al. Association of maternal inflammation during pregnancy with birth outcomes and infant growth among women with or without HIV in India. *JAMA Netw Open.* 2021;4(12):e2140584.
doi:10.1001/jamanetworkopen.2021.40584

eMethods.

eFigure. Levels of Third Trimester Inflammatory Markers by Preterm Birth Status (N = 218)

eTable 1. Association of Inflammatory Markers With PTB and LBW (Multivariable Model II)

eTable 2. Characteristics of the Study Population During Their Third Trimester by Low Birth Weight Status (N = 213)

eTable 3. Association of Inflammatory Markers With LAZ, WAZ, and WLZ (Multivariable Model II)

This supplementary material has been provided by the authors to give readers additional information about their work.

© 2021 Shafiq M et al. *JAMA Network Open.*

eMethods.

Data Collection and Laboratory Procedures

We collected sociodemographic information and clinical data from study participants at enrollment. Gestational age of mothers was determined by early pregnancy ultrasound. Follow-up visits were at third trimester (for those enrolled in second trimester), delivery, 6 weeks, and 3, 6, and 12 months postpartum. Postpartum maternal visits were paired with infant visits. The participants' visits were study specific but were coordinated to be conducted after their scheduled antenatal follow-up or infant immunization visits for mothers' convenience. Gestational age at delivery, along with infant length and weight at each visit were recorded thereafter. The research nurses who collected these measurements were trained in infant length and weight measurement. Their measurements were observed for inter- and intra-observer variations and were retrained half yearly. Standard procedures and instruments, used in other NIH network studies, were used for this study.

© 2021 Shafiq M et al. *JAMA Network Open.*

eFigure. Levels of Third Trimester Inflammatory Markers by Preterm Birth Status (N = 218)

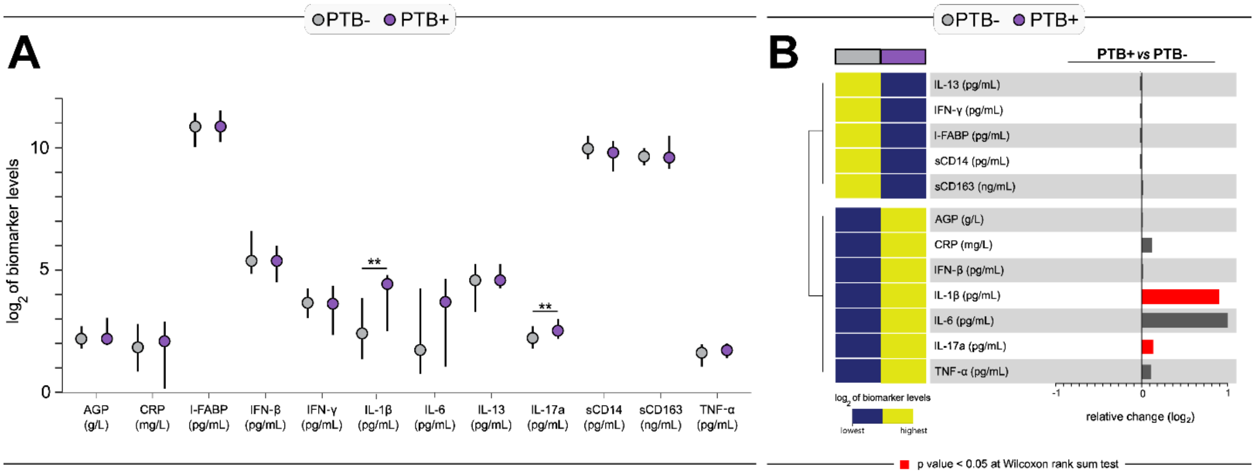

eFigure legend: (A) Median and interquartile range (IQR) log2 levels of markers, measured in the 3rd trimester is shown for women with PTB+ (n = 193) and PTB− (or term births) (n = 25). Wilcoxon rank-sum test was used to calculate *P* values. **P* < 0.05, ***P* < 0.01 and ****P* < 0.001. (B) Relative fold-change is shown for each marker by birth status. Red bars indicate *P* value <0.05.

© 2021 Shafiq M et al. *JAMA Network Open.*

eTable 1. Association of Inflammatory Markers With PTB and LBW (Multivariable Model II)

	**Multivariable Model II OR[a] (95% CI)**	
	**PTB[b]**	**LBW[c]**
**Log2 IFNB**	0.91 (0.73 - 1.14)	0.92 (0.78 - 1.08)
**Log2 CRP**	1.07 (0.96 - 1.19)	0.96 (0.80 - 1.16)
**Log2 AGP**	1.44 (0.78 - 2.67)	1.22 (0.82 - 1.81)
**Log2 I-FABP**	1.02 (0.69 - 1.49)	0.92 (0.71 - 1.20)
**Log2 IFN-γ**	0.82 (0.57 - 1.18)	1.11 (0.92 - 1.33)
**Log2 IL-1β**	**1.52 (1.15 - 2.01)**	1.20 (0.99 - 1.44)
**Log2 sCD14**	0.78 (0.48 - 1.29)	0.71 (0.48 - 1.05)
**Log2 CD163**	1.56 (0.89 - 2.74)	1.38 (0.90 - 2.10)
**Log2 TNF-A**	0.98 (0.61 - 1.57)	1.34 (0.98 - 1.82)
**Log2 IL-6**	1.19 (0.94 - 1.49)	1.07 (0.91 - 1.25)
**Log2 IL-17A**	**2.36 (0.99 - 5.64)**	**1.96 (1.09 - 3.49)**
**Log2 IL-13**	1.14 (0.83 - 1.57)	1.15 (0.92 - 1.45)

eTable 2 legend: The odds of PTB and LBW per increase in Log_2 concentrations of each inflammation marker (third trimester) and 95% CI is shown in the table. Multivariable model II is shown here for both PTB and LBW. Multivariable model II adjusted for maternal age, mid-upper arm circumference, HIV status, parity, smoking, history of PTB, anemia, education, and latent TB infection.

[a]OR: Odds ratio

[b]PTB: Preterm birth

[c]LBW: Low birth weight

© 2021 Shafiq M et al. *JAMA Network Open.*

eTable 2. Characteristics of the Study Population During Their Third Trimester by Low Birth Weight Status (N = 213)

	**Overall N = 213 (100%)**	**LBW[a] N = 64 (30%)**	**Normal Weight N = 149 (70%)**	***P* value**
**Age (median, IQR[b])**	24 (21 - 27)	25 (21 - 27.5)	23 (21 - 26)	.10
**Monthly Income**				
**≤ Rs. 10,255**	70 (33)	23 (37)	47 (32)	.53
**> Rs. 10,255**	141 (67)	40 (63)	101 (68)	
**Education**				
**None to primary**	52 (28)	13 (20)	39 (26)	.29
**Middle school to high school**	135 (63)	40 (63)	95 (64)	
**Post-high school**	26 (12)	11 (17)	15 (10)	
**Mid-upper arm circumference**				
**< 23 cm**	59 (28)	25 (39)	34 (23)	**.05**
**23 - 30.5 cm**	140 (66)	36 (56)	104 (70)	
**> 30.5 cm**	14 (7)	3 (5)	11 (7)	
**Smoking status**				
**Yes**	26 (12)	8 (12)	18 (12)	.99
**No**	187 (88)	56 (88)	131 (88)	
**History of PTB[c]**				
**Yes**	18 (8)	7 (11)	11 (7)	.42
**No**	195 (92)	57 (89)	138 (93)	
**HIV**				
**Yes**	69 (32)	26 (41)	43 (29)	.11
**No**	144 (68)	38 (59)	106 (71)	

Supplementary Table 1 legend: Data are presented as number (%) of subjects unless otherwise stated. *P* values were calculated using Fisher's exact test for categorical variables and Wilcoxon rank-sum for continuous variables to determine the difference between LBW and normal weight infants.

[a]LBW: Low birth weight

[b]IQR: Interquartile range

[c]PTB: Preterm birth

© 2021 Shafiq M et al. *JAMA Network Open.*

eTable 3. Association of Inflammatory Markers With LAZ, WAZ, and WLZ (Multivariable Model II)

	**Multivariable Model II Estimate (95% CI)**		
	**LAZ[a]**	**WAZ[b]**	**WLZ[c]**
**Log2 IFNB**	-0.01 (-0.08 - 0.06)	-0.01 (-0.08 - 0.06)	0.01 (-0.06 - 0.07)
**Log2 CRP**	0.05 (-0.05 - 0.15)	**0.09 (0.004 - 0.18)**	**0.10 (0.02 - 0.19)**
**Log2 AGP**	-0.05 (-0.16 - 0.06)	0.01 (-0.10 - 0.12)	0.06 (-0.07 - 0.19)
**Log2 I-FABP**	-0.01 (-0.13 - 0.11)	0.06 (-0.06 - 0.18)	0.06 (-0.05 - 0.16)
**Log2 IFN-γ**	0.002 (-0.08 - 0.08)	-0.01 (-0.06 - 0.05)	0.02 (-0.04 - 0.07)
**Log2 IL-1β**	**-0.11 (-0.21 to -0.02)**	**-0.08 (-0.16 to -0.003)**	-0.04 (-0.12 - 0.05)
**Log2 sCD14**	0.14 (-0.05 - 0.33)	**0.20 (0.05 - 0.35)**	0.12 (-0.04 - 0.28)
**Log2 CD163**	-0.15 (-0.38 - 0.08)	-0.10 (-0.32 - 0.12)	0.02 (-0.18 - 0.22)
**Log2 TNF-A**	0.03 (-0.10 - 0.16)	0.01 (-0.10 - 0.13)	-0.01 (-0.13 - 0.10)
**Log2 IL-6**	-0.003 (-0.08 - 0.07)	-0.02 (-0.09 - 0.05)	-0.03 (-0.09 - 0.03)
**Log2 IL-17A**	-0.16 (-0.43 - 0.12)	-0.09 (-0.33 - 0.15)	0.03 (-0.19 - 0.26)
**Log2 IL-13**	-0.04 (-0.14 - 0.06)	-0.03 (-0.11 - 0.05)	-0.03 (-0.13 - 0.07)

eTable 3 legend: The average increase in LAZ, WAZ and WLZ over the time-points delivery, 6 weeks, and 3, 6, and 12 months postpartum, are shown per increase in Log 2 concentrations of each inflammation marker (third trimester) and 95% CI in the table. Multivariable model II is shown here. Multivariable model II adjusted for maternal age, mid-upper arm circumference, HIV status, parity, smoking, anemia, education, and latent TB infection.

[a]LAZ: Length-for-age z-score

[b]WAZ: Weight-for-age z-score

[c]WLZ: Weight-for-length z-score

© 2021 Shafiq M et al. *JAMA Network Open.*
